# Interference of Apoptosis by Hepatitis B Virus

**DOI:** 10.3390/v9080230

**Published:** 2017-08-18

**Authors:** Shaoli Lin, Yan-Jin Zhang

**Affiliations:** Molecular Virology Laboratory, VA-MD College of Veterinary Medicine and Maryland Pathogen Research Institute, University of Maryland, College Park, MD 20742, USA; lsl1990@umd.edu

**Keywords:** hepatitis B virus (HBV), apoptosis, hepatocellular carcinoma (HCC), X protein

## Abstract

Hepatitis B virus (HBV) causes liver diseases that have been a consistent problem for human health, leading to more than one million deaths every year worldwide. A large proportion of hepatocellular carcinoma (HCC) cases across the world are closely associated with chronic HBV infection. Apoptosis is a programmed cell death and is frequently altered in cancer development. HBV infection interferes with the apoptosis signaling to promote HCC progression and viral proliferation. The HBV-mediated alteration of apoptosis is achieved via interference with cellular signaling pathways and regulation of epigenetics. HBV X protein (HBX) plays a major role in the interference of apoptosis. There are conflicting reports on the HBV interference of apoptosis with the majority showing inhibition of and the rest reporting induction of apoptosis. In this review, we described recent studies on the mechanisms of the HBV interference with the apoptosis signaling during the virus infection and provided perspective.

## 1. Introduction

Hepatitis B virus (HBV) is an enveloped DNA virus with a reverse transcription phase, belonging to the *Orthohepadnavirus* genus, the *Hepadnaviridae* family [1,2]. The genome of HBV is only 3.2 kb, containing four overlapping open reading frames (ORFs). The four ORFs encode seven viral proteins: pre-S1, pre-S2, S, pre-C, C, viral polymerase, and HBV X protein (HBX). There are four regulatory elements in the genome: enhancer II/basal core promoter, pre-S1 promoter, pre-S2/S promoter, and enhancer I/X promoter. The core protein and the viral polymerase are translated from the pre-genomic RNA (pgRNA), while the regulatory HBX protein and the three envelope proteins are encoded by the subgenomic RNAs [1,2]. The HBV virions attach to host cells through heparan sulfate proteoglycans or the hepatocyte-specific pre-S1 receptor, sodium taurocholate cotransporting polypeptide (NTCP) [3]. The virions enter the cells by endocytosis or fusion of the viral envelope at the plasma membrane. Once entering the cells, the viral nucleocapsid containing the partially double-stranded DNA, known as the relaxed circular DNA (rcDNA), would be released into the cytoplasm and transported into the nucleus [2]. The plus strand of the rcDNA is repaired and completed by the viral polymerase in the nucleus to generate the covalently closed circular DNA (cccDNA), which is transcribed into RNAs for the viral replication. The HBV DNA can be integrated into the host genome and the integration is commonly seen in patients with hepatocellular carcinoma (HCC).

HBV infection causes both acute and chronic liver diseases, and accounts for most of the chronic liver diseases globally, affecting over 240 million people worldwide [4]. More than one million individuals die from cirrhosis and liver cancer caused by the chronic HBV infection each year [5]. HBV infection is predominantly prevalent in Asian countries, such as China, Japan, Taiwan, and Korea [6]. In the USA, HBV infection is most common among Asians [7]. Up to now, ten genotypes (A–J) of HBV have been identified [8]. Genotypes A and D are ubiquitous but prevalent in Europe and Africa, while genotypes B and C are confined in Asia and Oceania. Other genotypes (E–J) are occasionally observed in some Asian countries. The virus can be transmitted through blood, semen, and body fluid, or from mother to baby at birth [9,10]. For some people, hepatitis B is an acute or short-term illness; but for others, it can become a long-term, chronic infection [11,12]. This indicates that HBV infection is just a trigger for liver diseases and HCC development, which are possibly the consequence of a complex interplay of many factors including host immune response. There is a large proportion of HBV-inactive carriers, who present little virus replication, normal alanine aminotransferase (ATL) level and minimal liver inflammation [13]. However, some of them may undergo HBV reactivation when treated with immunosuppressive drugs or suffer a higher risk of hepatocellular carcinoma after excessive alcohol consumption [14,15]. Despite an effective vaccine for prevention, there is still no known cure for existing HBV infection.

The frequent integration (>70%) of HBV DNA into the cell genome contributes to the instability of the chromosome, interruption of key cellular pathways and mutation of some pro-cancer genes [16]. HCC progression is found to associate with the patient ages and the HBV genotypes. The HBV genotype A mainly causes acute liver disease in clinical settings. An epidemiological investigation in China shows that, in young HCC patients (<30 years old), the HBV B2 is predominant, and a breakpoint in chromosome 8q24 located between *c-Myc* and plasmacytoma variant translocation 1 (*PVT1*) is more frequently found than in older patients. HBV integration into this site leads to the overexpression of *c-Myc* and *PVT1*, and consequent HCC progression [17]. Aside from the *HBV B2* genotype, the *HBV C* genotype also accounts for a large number of clinical HCC cases [8].

Chronic HBV infection is often accompanied by HCC. Among all the cancer cases caused by the infectious agents, 19.2% are attributed to HBV, while 7.8% are caused by hepatitis C virus (HCV) [18]. Some tumor diseases or HCC are frequently accompanied by defective apoptosis. In clinically histochemical staining of human carcinoma tissues, the Fas-expression is much lower than their corresponding non-carcinoma tissues, in both frequency and amount. Moreover, the apoptotic cell percentage is lower in the Fas-defective tissues [19]. During virus infection, the host cells take some protective measures such as cell death to prevent virus replication or dissemination [20]. To survive in the host cells, the viruses have evolved various mechanisms to modulate the apoptosis signaling during infection. They can promote the cell apoptosis and fission to facilitate the virus dissemination, or antagonize the apoptosis to gain time to proliferate in the infected cells. For example, HCV accelerates the cell apoptosis by the activation of caspase 3 and the release of cytochrome C, whereas Myxoma virus produces viral B-cell lymphoma 2 (BCL-2) to prevent the cell apoptosis [21]. In addition, some oncogenes are also upregulated during virus infection, such as HBV and avian leukemia virus [22,23].

HBX, approximately 17.4 kDa, is a viral protein with multiple functions. This protein can be translated in host cells from the integrated HBV genome even in the absence of complete virus replication cycle [24,25]. Thus far, the function of X protein is the most widely studied among all the HBV proteins. The small regulatory protein is implicated to play a major role in HCC progression [26,27,28]. It is able to not only suppress the DNA repair machinery [29,30] but also affect the DNA methylation of the host cells. These activities might contribute to the HCC progression [31,32]. HBX has also been demonstrated to have interplay with non-coding RNA (ncRNA) and various signaling pathways to regulate the host cell activities. Since the cell apoptosis is highly correlated with the HCC progress, understanding how the virus interferes with the apoptotic process may shed light on the mechanism of HCC formation and facilitate the development of anti-tumor therapeutics. Here, we summarize recent studies on the mechanisms of HBV interference of the apoptosis.

## 2. Apoptosis

Apoptosis is a programmed cell death, which is highly organized and acts as a protective strategy for healthy organisms to maintain the homeostasis [33]. The apoptosis plays a vital role in the innate and adaptive immune responses. The morphology of apoptotic cells is featured by cell shrinkage, membrane blebbing and the formation of apoptotic bodies [34,35,36]. Apoptotic cells do not release their cellular contents to the surroundings before being phagocytosed in vivo. The predominant pathways of the apoptosis are mainly composed of the extrinsic pathway (death receptor pathway) and the intrinsic pathway (mitochondrial pathway) [37].

**Figure 1:**
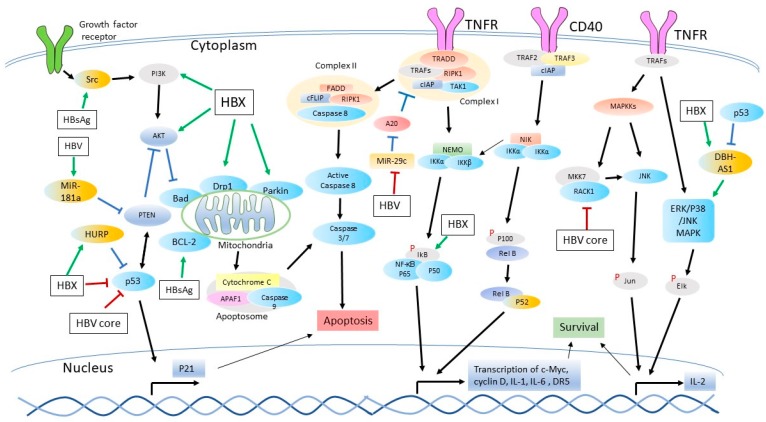
Inhibition of apoptosis by hepatitis B Virus (HBV) infection. Hepatitis B Virus X protein (HBX) and HBV core inhibit p53-mediated apoptosis. HBX activates the phosphatidylinositol-4,5-bisphosphate 3-kinase-protein kinase B (PI3K-Akt) pathway to inhibit apoptosis via the upregulation of PI3K and the induction of Akt phosphorylation. HBX inhibits the intrinsic apoptotic pathway by recruitment of Drp-1 and Parkin to the mitochondria for mitochondrial fission and mitophagy. The activation of Akt also prevents translocation of BAD to the mitochondria, thereby preventing apoptosis. HBX can activate the nuclear factor kappa-light-chain-enhancer of activated B cells (NF-κB) signaling via the degradation of IκB. In the MAPK-JNK pathway, HBV can attenuate the function of the kinase that activates JNK. HBV can downregulate apoptosis by either elevation of anti-apoptotic ncRNAs such as MiR-181a, or decrease of pro-apoptotic ncRNAs, such as MiR-29c. Green arrows next to the white boxes (HBV or HBV proteins) denote the activation of apoptosis. Red bars to the white boxes stand for the inhibition of apoptosis. Drp-1: Dynamin-1-like protein. BAD: BCL-2-associated death promoter. MAPK: MAP kinase. JNK: c-Jun N-terminal kinases. ncRNA: Non-coding RNA. MiR-181a/29c: MicroRNA 181a/29c.

The extrinsic pathway is mostly triggered by the external stimuli such as Fas, tumor necrosis factor-α (TNF-α), TRAIL (TNF-related apoptosis-inducing ligand), APO3L and APO2L [37]. Binding of the extracellular ligands to their receptors on the cell surface leads to the recruitment of adaptor proteins to transmit the intracellular signals via the caspase cascades. The recruited caspase 8422 and Fas-associated protein with death domain (FADD) forms an oligomeric death-inducing signaling complex (DISC), leading to the cleavage and activation of caspase 8. The activated caspase 8 then cleaves and activates the effector caspase 3/7, which are able to cleave a broad spectrum of cellular targets, such as receptor-interacting protein (RIP), X-linked inhibitor of apoptosis protein (X-IAP), signal transducer and activator of transcription-1 (STAT1), topoisomerase I, vimentin, retinoblastoma (Rb), and lamin B, consequently resulting in the cell death [38,39,40]. For the TNF receptor (TNFR), upon activation, TNFR recruits adaptor proteins TNFR type 1-associated DEATH domain protein (TRADD), Fas-associated protein with death domain (FADD), receptor-interacting serine/threonine-protein kinase 1 (RIPK1), cellular inhibitors of apoptosis (cIAP), TNF receptor associated factors 2 (TRAF2), and TRAF5. These proteins form the complex I (Figure 1), which leads to initiation of the canonical nuclear factor kappa-light-chain-enhancer of activated B cells (NF-κB) pathway [41]. In the complex I, IAPs can exert an anti-apoptotic role in the cells, for instance, cIAP1/2 interact with the second mitochondrial activator of caspases (SMAC) and sequester it from the XIAP, and the released XIAP inhibits caspases and apoptosis [42,43]. However, the anti-apoptotic role of cIAP1 can be counteracted by the SMAC mimetics [44]. Under certain circumstances, such as loss of cIAP, a secondary death promoting complex, termed as the complex II, can be formed. The complex II is composed of TRADD, FADD, RIPK1, caspase 8, and is capable of inducing apoptosis or necroptosis [45,46,47]. Notably, the complex II-initiated apoptosis can be prevented by cellular FLICE-inhibitory protein (cFLIP) through the inhibition of caspase 8 activity [48]. The expression of cFLIP is mediated by the NF-κB signaling.

The intrinsic pathway is initiated by the internal stimuli, such as DNA damage, endoplasmic reticulum (ER) stress, hypoxia and metabolic stress [37]. In healthy cells, the BCL-2 sequesters its proapoptotic counterparts, BAX (BCL-2-associated X protein), BAK (BCL-2 homologous antagonist/killer), and BCL-2 homology domain 3 (BH3)-only proteins into inactive complexes. Under cell stress, BH3-only proteins are activated, followed by the release of BAK/BAX and the engagement of the homo-oligomerization of the two proteins. The self-association of BAK/BAX is inclined to form a lipidic pore in the outer membrane (OM) of mitochondria by inserting α-helices 5 and 6 of the dimer into the OM, resulting in mitochondrial outer membrane permeabilization (MOMP) [49,50]. The MOMP allows inner mitochondrial proteins, such as apoptosis inducing factor (AIF), SMAC and cytochrome C, to be released into the cytosol. While SMAC exerts its pro-apoptotic role through binding with cIAPs, cytochrome C interacts with apoptotic protease activating factor 1 (APAF1) to facilitate the formation of the apoptosome. Once formed, the apoptosome will then recruit and activate the pro-caspase 9. The cleaved pro-caspase 9 becomes active and then activates the caspase 3/7, culminating in the cell apoptosis [51,52].

In addition to the caspase-dependent intrinsic pathway, the AIF protein can induce apoptosis by triggering chromatin condensation and DNA fragmentation, independent of caspase activation. After being released from the mitochondria, AIF ends up in the nucleus where it signals the cell to chromosome condensation and DNA fragmentation by Ca^2+^ and Mg^2+^-dependent endonucleases [53,54].

The extrinsic pathway and the intrinsic pathway have some crosstalk via protein BH3 interacting-domain death agonist (BID). The activated caspase 8 is demonstrated to cleave BID, which belongs to the “BH3-domain-only” subset of the BCL-2 family. The truncated BID (tBID) triggers BAK or BAX homo-oligomerization and consequently MOMP [55]. In the process of apoptosis, the level of reactive oxygen species (ROS) plays an important role in deciding the cell fate. It interferes with both the extrinsic and the intrinsic pathways. Low-level ROS promotes the cell survival signaling, while toxic level ROS induces cell apoptosis [56]. The cancer cells usually have higher level ROS and are more capable to scavenge excessive ROS than the normal cells [57]. The higher level of ROS enhances cell proliferation through inducing abnormal cell growth caused by genetic mutation, enhanced autophagy, or activation of various signaling pathways, such as the phosphatidylinositol-4,5-bisphosphate 3-kinase-protein kinase B (PI3K-Akt) pathway, NF-κB, and protein kinase D (PKD) pathway. The toxic level of ROS leads to the apoptotic death of cancer cells.

Apart from the two extensively studied pathways, there are the physiological pathway, the perforin/granzyme pathway, and the pathological apoptosis pathway. In order to maintain the homeostasis of the human body, numerous cells need to be sacrificed every day to balance the metabolism. For instance, during the development of immune system, most lymphocytes lacking B cell receptor (BCR) or T cell receptor (TCR) will be eliminated by selection process [58]. Some diseases also cause excessive apoptosis in human tissue. A classic example is that the human immunodeficiency virus (HIV) Tat protein increases the Fas expression of CD4^+^ T cells, increasing the possibility of T cell elimination [59]. The perforin/granzyme B pathway is one of the mechanisms that the cytotoxic T lymphocytes (CTL) and natural killer (NK) cells utilize to kill their target cells. The perforin forms poly-perforin pores on the target cell membrane, inducing the osmotic instability and allowing granzyme B to pass through the cell membrane to cause cell lysis. This mechanism can lead to cell death in the absence or presence of the activated caspases [60].

## 3. Hepatitis B Virus and Apoptosis

Plenty of studies have been performed to determine the relationship of HBV infection and apoptosis, but the results are still contradictory. The majority of the papers showed that HBV or HBX could inhibit the cellular apoptosis, thereby facilitating the virus proliferation and promoting the HCC progression [61,62,63]. Various studies have been done to define the balance among the HBV proliferation, apoptosis and HCC. For long-term persistence in the host cells, HBV may inhibit cell death by either activating oncogenes or disrupting signaling pathways, thereby promoting the HCC progression. HBV can also inhibit apoptosis and promote HCC development through the upregulation of some pro-growth proteins, such as cationic amino acid transporter 1 (CAT-1) [64]. In some clinical cases, the CTL response is also relatively weak in chronic HBV patients, culminating in apoptosis of a smaller proportion of infected hepatocytes [65,66]. In chronical HBV-infected mice, cIAPs restrict the TNF-mediated HBV elimination as well as HBV-infected hepatocytes death, while the inhibition of cIAPs by SMAC mimetics or silence of cIAPs boosts HBV clearance in the presence of TNF and HBV-specific CD4^+^ T cells [67,68]. On the other hand, HBV induction of apoptosis is also reported in some papers [69,70,71,72,73,74]. The reason for the discrepant results of HBV effect on apoptosis is not known, but possibly due to the different experimental conditions or the HBV genotypes used in the different laboratories.

### 3.1. Inhibition of Apoptosis by Hepatitis B Virus

Among the HBV proteins, HBX is the most frequently reported one to be associated with the inhibition of apoptosis and the activation of HCC progression. It may block apoptosis through the sequestration of cytoplasmic p53, activation of PI3K-Akt pathway, inhibition of death receptor mediated apoptotic pathway, activation of NF-κB signaling pathway, inhibition of mitochondrial apoptotic pathway, as well as interplay with ncRNA [75,76,77,78,79,80]. The mechanisms of HBX inhibition of apoptosis are summarized below (Figure 1).

#### 3.1.1. Sequestration of p53 Signaling

P53, a tumor suppressor, is not only a transcription factor that regulates the expression of a variety of genes but also induces apoptosis [81,82]. A Japanese research group shows that by expressing HBX in HepG2 and HLF cells with Cre/Lox system, apoptosis is induced independently of p53 [83]. In contrast, HBX is demonstrated to be capable of abolishing p53-induced apoptosis by binding it (Figure 1) [75,84]. Mutations of nucleotides, A1762T and G1764A, of HBX are frequently reported in HBV isolated from chronically infected patients. The mutant HBX promotes the replication of HBV (subtype *adw*2), resulting in a higher viral load in hepatoma cells [85,86]. In HepG2.2.15 cells, the mutant HBX binds to p53 and blocks its downstream gene transcription, while the wild type HBX (subtype *ayw*) only binds to p53 without affecting the p53-mediated transcription [87]. In addition, the restriction of p53 signaling by HBX may vary in different cell types. In primary human hepatocytes, the wild type HBX (genotypes *ayw* and *adr*) is able to quench p53 in the cytoplasm, and the C terminal portion of HBX is responsible for the sequestration [88,89]. In contrast, in HepG2 and Hep3B tumor cells, HBX is pro-apoptotic and enhances the nuclear translocation of p53 through the activation of ATM kinase, which phosphorylates p53. However, in a later study, the inhibition of p53 by HBX could only be achieved when p53 is expressed at a relatively high level in the cells (HepG2, Hep3B and NIH/3T3 cells) [90]. The hepatoma upregulated protein (HURP), a cellular oncogene that mediates the degradation of p53, is upregulated by HBX in HCC, and consequently, inhibits the cisplatin-induced apoptosis [62]. Although the HBX and p53 interaction is reported, transduction of HBX in transgenic mice indicates that this interaction is not sufficient for tumor formation [91]. Therefore, the HBV-mediated inhibition of apoptosis through the interference of p53 may be dependent on cell types, experimental models, HBX structure and p53 level.

#### 3.1.2. Activation of PI3K Pathway

In many cancers, the PI3K-Akt pathway is overactive, thus reducing apoptosis and allowing cell proliferation (Figure 1) [92]. Akt is normally considered as an anti-apoptotic protein through antagonizing pro-apoptotic proteins or facilitating the induction of other anti-apoptotic proteins [93,94,95,96]. In HBX-overexpressed Hep3B cells and 293T cells, Akt is activated to phosphorylate IκB kinase (IKKα) and promote its nuclear translocation, which is found to promote cell migration and invasion [97]. In Chang liver cells, HBX activates the PI3K-Akt, leading to the phosphorylation and blockade of BCL-2-associated death promoter (BAD), a pro-apoptotic protein inducing mitochondrial permeability transition pore (MPTP). Consequently, the cytochrome C release and apoptosis are prevented [98]. In a human placental trophoblastic cell line, HBX inhibits apoptosis via the elevation of PI3K expression to strengthen the activity of the PI3K-Akt pathway [99]. In addition, the transforming growth factor beta (TGF-β)-induced apoptosis in Hep3B cells can be rescued by the HBX-activated PI3K-Akt pathway [76]. A previous study shows that the anti-apoptotic effect of HBX is dependent on its isoforms [100]. The HBX isoform that contains the Akt phosphorylation site at Ser31 functions as an anti-apoptotic protein. This isoform can be phosphorylated by Akt and in turn activate the PI3K-Akt pathway. In contrast, the isoform that does not contain the Akt phosphorylation site plays an opposite function in apoptosis [100].

#### 3.1.3. Inhibition of the Death Receptor-Mediated Apoptotic Pathway

In the extrinsic apoptotic pathway, HBX potently inhibits the caspase 3 activity [79,101]. HBX has been shown to inhibit the Fas-induced apoptosis, and this process is independent of p53 [79]. In this study, HBX transfection rate in primary hepatocytes is significantly enhanced from 5% to 80% by co-expressing HBX and enhanced green fluorescence protein (EGFP). Simultaneously, HBX inhibited the activation of caspase 8 and 3 and the release of cytochrome C. The HBX expression is associated with the upregulation of SAPK/JNK signaling, and furthermore, the ^26^RXRXXS motif of HBX is essential for the SAPK upregulation and the inhibition of Fas-mediated cell killing. HBX induces the activation of NF-κB signaling via the degradation of inhibitor of kappa B (IκB), which also contributes to the inhibition of Fas-induced apoptosis (Figure 1) [63,102].

#### 3.1.4. The Activation of NF-κB Pathway

NF-κB is generally regarded as a positive regulator of cell growth [103,104,105]. The NF-κB signaling consists of the canonical and the non-canonical NF-κB signaling pathways (Figure 1). The canonical NF-κB signaling is initiated through receptors such as toll-like receptors (TLRs), tumor necrosis factor receptor (TNFR), T-cell receptor (TCR) or B cell receptor (BCR). The receptor-mediated activation of transforming growth factor beta-activated kinase 1 (TAK1) phosphorylates IKK complex, which consequently degrades IκB, leading to the release of NF-κB heterodimer (p65/p50) into the nucleus. The non-canonical pathway is triggered by a signaling from a subset of TNFR members, such as B cell activating factor receptor (BAFFR), CD40, lymphotoxin β-receptor (LTβT) and receptor activator for nuclear factor κB (RANK). Through the activation of NF-kappa-B-inducing kinase (NIK) and IKKα, p100 is processed into the active p52, which forms a heterodimer with *RelB*. The subsequent nuclear translocation of the RelB/p52 results in a persistent stimulation of the pathway [106]. Moreover, the accumulation of NIK is reported to activate the canonical NF-κB pathway through the enhancement of IKK complex activity [107]. In the process, the anti-apoptotic protein IAPs can both positively and negatively regulate the canonical or the non-canonical NF-κB signaling. For instance, upon engagement of TNFR (see Section 2), the cIAP in complex I promotes the ubiquitination of RIPK1, which leads to the activation of TAK1 [108]. In addition, the ubiquitinated RIPK1 can prevent apoptosis by suppressing the formation of complex II and the activation of caspase 8. In contrast, IAPs also exert an inhibitory role in the NF-κB pathways. For example, the basal level of NIK activation is very low due to the control of upstream TRAF3-TRAF2-cIAP complex, and cIAP1/2 degrades NIK by ubiquitinating the protein, while the loss of any component of the complex leads to an accumulation of NIK and the activation of both NF-κB signaling pathways [107,109,110,111].

The NF-κB signaling is constitutively activated in many cancers, and the NF-κB activation contributes to tumorigenesis [112,113]. There are several mechanisms that NF-κB antagonizes cell death. First, the NF-κB activation leads to an elevation of anti-apoptotic genes. Secondly, NF-κB induces the production of immune response cytokines, such as TNF-α, IL-1 (Interleukin-1), IL-6, and IL-8. Moreover, NF-κB induces the expression of some pro-oncogenic genes, such as *cyclin D1*, *c-Myc*, and cIAPs [114]. In addition, the NF-κB signaling contributes to tumor progression by facilitating epithelial to mesenchymal transition and metastasis, as well as aiding the vascularization of tumors via the upregulation of vascular endothelial growth factor (VEGF) [115,116,117]. The activation of NF-κB signaling increases the stability of HBX protein [118]. Several studies have demonstrated that HBV infection leads to the activation of NF-κB, followed by the inhibition of apoptosis and increase of cell progression (Figure 1) [119,120,121,122,123,124]. HBX induces the activation of NF-κB by degrading IκB [119,120]. IκB is responsible for sequestering NF-κB in the cytoplasm. Once IκB is phosphorylated and degraded, the NF-κB heterodimer translocates into the nucleus and initiates the transcription of downstream genes. To examine the correlation between NF-κB activation and apoptosis, IκB-SR, an isoform that cannot be phosphorylated, is introduced into NIH/3T3 cells. The cotransfection of IκB-SR and HBX results in increased apoptosis [121]. In the presence of IκB-SR, HBX overexpression induces MPTP. Notably, in the context of HBV replication, HBX activates NF-κB and inhibits the cytochrome C release from the mitochondria. However, when the NF-κB activity is inhibited, the HBX in the context of HBV replication could induce apoptosis through MPTP. This study indicates that, depending on the status of NF-κB activity, HBX can be either pro- or anti-apoptotic [122]. In the HBV-positive cell line, HepG2.2.15, the cIAP1 and cIAP2 are expressed much higher than in HepG2, indicating that HBV replication might boost the anti-apoptotic proteins [78]. In addition, the activation of NF-κB is found to initiate enhanced transcription of both anti-apoptotic genes such as gp96, survivin, p21 and the pro-apoptotic genes such as death receptor 5 (*DR5*) [123]. The expression of the anti-apoptotic genes may be responsible for the multidrug resistance of HBX-transfected HepG2 cells [124]. On the other hand, the upregulation of death domain receptor accounts for the increased sensitivity of cells to apoptotic stimuli. In different cell lines, the regulation effect of HBX also varies [125]. To illustrate this point, two HBX-expressing stable cell lines were established, namely, Huh-7-X and CHANG-X. The mRNA levels of p21, p27, and TGF-β are drastically downregulated in Huh-7-X stable cells but have a minimum change in CHANG-X stable cells [125]. Collectively, the NF-κB signaling is important not only in the innate immune system but also in the release of cell stress and the promotion of hepatocytes growth, as well as in the regulation of apoptosis upon HBV infection.

#### 3.1.5. Inhibition of the Mitochondria-Mediated Apoptotic Pathway

Sequence analysis from tumor tissues and para-tumor tissues of 47 patients shows a combination of mutations (10Ala/Arg and 144Ser/Arg) exists in HBX with high frequency [61]. HBX harboring these two mutations reduces BAX expression and inhibits apoptosis in HepG2 cells. HBX is also able to inhibit serum-starvation induced mitochondrial apoptosis via the activation of autophagy, which is featured by increased microtubule-associated proteins 1A/1B light chain 3B (LC3II) and Beclin-1 [126,127]. As a core component of PI3K-III complex, Beclin-1 plays an important role in autophagy and cell death [128], while the interaction between BCL-2 and Beclin-1 does not counteract the anti-apoptotic role of BCL-2 [129]. HBX can sequester AIF, a caspase-independent protein in the intrinsic pathway, in the cytoplasm, resulting in the prevention of DNA fragmentation and apoptosis [130]. In Huh-7 cells, HBV and HBX can disrupt mitochondrial dynamics by inducing the translocation of dynamin-related protein Drp-1 to the mitochondria and the subsequent mitochondrial fission [77]. Parkin, an E3 ligase, is also translocated to the mitochondria and associated with the mitophagosome triggered by HBV/HBX. Parkin expression is upregulated in the presence of HBV/HBX. The enhanced Parkin level promotes the mitophagy, which attenuates apoptosis. Silencing of Parkin induces the mitochondrial apoptotic signaling. Thus, HBV promotes aberrant mitochondrial dynamics to protect cells from apoptosis in HepAD38 cells [77]. In chronic HBV-infected patients, the mitochondrial polarization in CD8^+^ T cells is impaired, and a higher level of ROS is detected in chronic patients in comparison with healthy individuals [131]. Further, the restoration of mitochondrial function via mitochondria-targeted antioxidants reactivates the exhausted T cells and helps with HBV clearance in the chronic HBV patients [131].

#### 3.1.6. Interference of Apoptosis through ncRNA

NcRNA accounts for 90% of genomic RNA, and it can be divided into the long non-coding RNA (lncRNA) and the microRNA (miRNA or MiR hereafter) [132]. Recently, increasing studies show that ncRNA has essential biological functions such as modulating cell proliferation, cell cycle, apoptosis, invasion and metastasis in cancers [133]. The interaction of HBV and ncRNA has also been widely studied. HBV and HBX inhibit the cell apoptosis by the interference of ncRNA. This is supported by the observation that HBV or HBX-transfected HepG2 cells have significantly upregulated MiR-181a and decreased PTEN, a tumor suppressor protein. PTEN inhibits PI3K-Akt and protects p53 by attenuating the mouse double minute 2 homolog (Mdm2) translocation into the nucleus (Figure 1) [134]. Upregulation of miR-181a suppresses PTEN expression, and inhibition of miR-181a abolishes the inhibitory effect of HBX on PTEN protein [135]. A novel lncRNA DBH-AS1 has been shown to activate ERK/p38/JNK MAPK (extracellular signal-regulated kinases/p38/ c-Jun N-terminal kinases mitogen-activated protein kinase) signaling and promote cell proliferation. HBX promotes the generation of DBH-AS1, thereby inhibiting serum starvation-induced apoptosis in HCC [136]. MiR-221, promoting cell proliferation by suppression of estrogen receptor-α, is also obviously increased in HBX-transfected HCC cells [137]. Aside from the function of HBV proteins, HBV transcripts in transgenic mice absorb the MiR-15a/16 and increase expression of the anti-apoptotic proteins BCL-2 and Smad7 [138,139].

In HBV-transfected HCC cell lines and clinical tumor tissues, pro-apoptotic microRNA MiR-29c is significantly downregulated [80]. The MiR-29c inhibits cell proliferation through suppressing A20, an E3 ligase negatively regulating NF-κB signaling and TNF-induced apoptosis via downregulating the E3 ligase activity of TRAF2 and TRAF6 (Figure 1) [140]. In HBV-related HCC patients, the MiR-122 and MiR-22 are significantly lower than those in benign liver diseases and non-HBV-related HCC patients, underlying that the miRNAs play vital roles in the HBV-related HCC formation [141]. These data suggested that ncRNA could possibly play an important role in the regulation of cell progression, either positively or negatively, while HBV may interfere with cell apoptosis through the modulation of those ncRNAs. The research progress on the interplay between ncRNA and apoptosis during HBV infection is recently reviewed in detail by Zhang et al. [142].

#### 3.1.7. Other Inhibitory Pathways

In addition to the signaling pathways described above, HBV also inhibits apoptosis by the upregulation of pro-oncogenesis genes or the activation of cell progression pathway. For instance, cell division control protein 42 homolog (CDC42), a member of the Rho GTPase family, is known to facilitate tumorigenesis and cancer progression. It is upregulated in HBX-overexpressed Huh-7 cells, resulting in higher cell proliferation and reduced apoptosis [143]. Manganese superoxide dismutase (MnSOD) is responsible for scavenging superoxide anion and preventing cells from DNA damage. HBV infection increases the expression of MnSOD, which is mediated by HBX protein [144]. Notch signaling and Smad pathway promote cell proliferation, while, in HCC and HTR-8/SVneo cells, HBX expression activates these pathways to suppress apoptosis [145,146].

### 3.2. Pro-Apoptotic Effect of HBV and HBX

**Figure 2:**
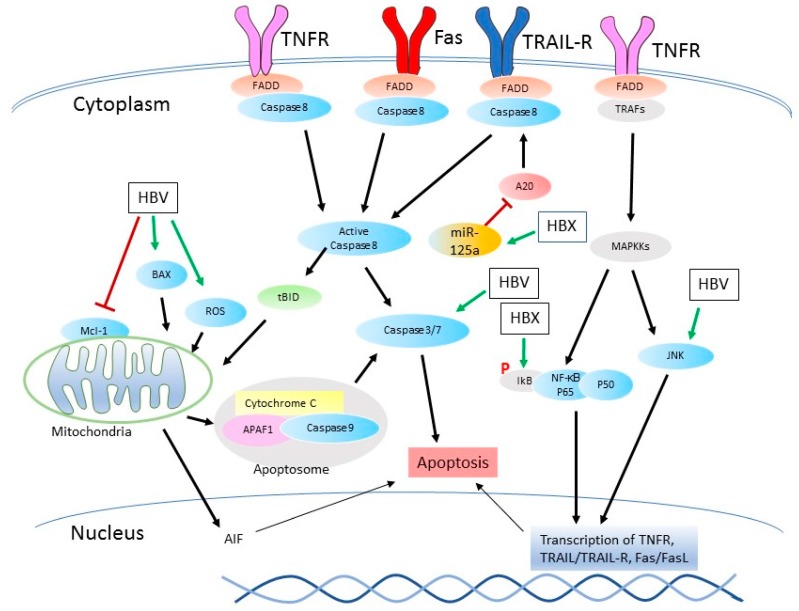
Pro-apoptotic role of HBV. HBV infection causes the activation of NF-κB in hepatoma cells, and subsequent excessive expression of the death-associated receptors, which increase the cell sensitivity to stimuli. In addition, HBV directly induces the cleavage of caspase 3 to activate apoptosis. HBV induces the mitochondrial apoptotic signaling pathway by increasing the BAX expression and the ROS level or downregulating Mcl-1. The BCL-2 homology domain 3 (BH3)-like domain in HBX also plays a role in the induction of apoptosis. Green arrows mean the activation step of apoptosis; Red bars stand for the inhibition step of apoptosis.

Although the suppression of apoptosis contributes to the progression of carcinogenesis, apoptosis can still be observed in untreated malignant tumors [147]. The apoptosis could be induced by CTL in tumor tissue or could be activated by TNF-α treatment [72,148]. HBV can also activate apoptosis or sensitize host cells to apoptosis induction in in vitro studies, through the direct activation of apoptotic proteins, regulation of Ca^2+^ concentration or the upregulation of cell death receptors [149,150]. Following are signaling pathways that HBV interrupts to induce cell apoptosis (Figure 2).

#### 3.2.1. Death Receptor-Mediated Signaling Pathways

HBV has been reported to induce apoptosis in liver biopsies of HBV patients [151]. The virus infection in transgenic mice and hepatocytes increases the cell sensibility to TRAIL-induced apoptosis by increasing the expression of BAX (Figure 2) [73]. HBX expression in hepatocytes has the same outcome as HBV infection. In clinical HBV liver samples, the TRAIL expression in chronic hepatitis B samples is the highest in comparison to acute hepatitis B samples, liver cirrhosis, and normal liver samples. This result suggests that TRAIL expression may have some correlation with the extent of liver injury [152]. Further studies show that the death receptor TRAIL-R5 expression is enhanced by HBX in Huh-7 cells through the activation of the NF-κB pathway, which contributes to the increased apoptosis induced by TRAIL [74]. Although A20 is upregulated in HBV-infected HCC cells, liver tissue, and serum of chronic HBV-infected patients [80,153], HBX overexpression in hepatocytes leads to A20 reduction [154]. HBX sensitizes the hepatocytes to the TRAIL-induced apoptosis by repressing the A20 expression and its ubiquitin ligase activity (Figure 2) [154].

In addition to TRAIL, HBX is also able to sensitize cells to the TNF-α-induced apoptosis through the activation of MAP kinase kinase kinase/c-Jun N-terminal kinases (MEKK/JNK) signaling pathway and the nuclear accumulation of N-Myc [72], or by decreasing the expression of Bcl-xL [155].

Another death receptor, Fas, and its ligand FasL are upregulated in rat renal tubular epithelial cells (NRK-52E) transfected with HBX, and this increase is due to the activation of the MLK3-MKK7-JNK pathway [156]. The Fas sensitivity is reconstituted in HBX transgenic mice through the decrease of BCL-2, despite no direct interaction between HBX and BCL-2 family members [70]. Another group demonstrates that HBX activates the p38 MAP kinase and JNK pathways, inducing the transcription of Fas/FasL and TNFR1/TNF-α. The increased expression of death receptors induces the cleavage of pro-caspase 8, with subsequent tBID activation and cytochrome C release [150]. In addition, a recently identified novel ORF of HBV, HBwX that fuses HBX and its upstream 56 amino acid residues, has been shown to sensitize HCC cells to the adriamycin (ADM) and LPS-induced apoptosis [157].

#### 3.2.2. The Mitochondria-Mediated Cell Death

The interaction between HBV and the mitochondria-mediated apoptosis is extensively studied. HBX overexpression contributes to the aggregation of the mitochondria, leading to cell death [158]. In the same study, HBX was found to colocalize with p53, but this association has no correlation with mitochondrial aggregation, suggesting two independent mechanisms in apoptosis induction. HBX binds to BAX in HepG2 cells, leading to enhanced translocation to the mitochondria, followed by loss of mitochondrial membrane potential [71]. Aside from the pathways mentioned above, HBV also sensitizes HL7702 cells to the oxidative stress-induced apoptosis through increasing the opening of MPTP [159]. The Mcl-1, a member of the anti-apoptotic BCL-2 family, is drastically declined during this process (Figure 2) [160].

BH3-like protein is an important initiator of the mitochondrial apoptotic pathway. Both HBX and a spliced viral protein, HBSP, have BH3 domain. Both proteins induce caspase 3 dependent apoptosis in HepG2 cells, while an amino acid mutation in the BH3 domain results in loss of the capability to induce apoptosis [161]. Possibly due to mutations in the BH3 domain of genotypes A and C of HBV, the two genotypes have weaker pro-apoptotic activity than genotype B in HepG2 cells [162]. Further study shows that HBX causes apoptosis in *Caenorhabditis elegans* by targeting BCL-2 homolog protein CED9, through the interaction between the BH3 domain and CED9 [163]. However, a recent structural and biochemistry analysis presents an opposite result, that is, the interaction between BCL-2 and HBX BH3-like domain is much weaker than the canonical BH3 and BCL-2 [164], indicating that the mechanism of HBX BH3 motif interfering with BCL-2 might be different. In addition, HBV has been shown to induce oxidative stress, accompanied by increased ROS level [165].

### 3.3. The Roles of the Other HBV Proteins

Aside from HBX protein, the other HBV proteins also have roles in cell apoptosis, either positive or negative. For example, HBsAg prevents the translocation through interaction with jumping translocation breakpoint protein (JTB), thereby inhibiting cell apoptosis mediated by JTB [166]. The large HBsAg glycoprotein inhibits apoptosis by activating the Src/PI3K/Akt pathway through the activation of Src kinase (Figure 1) [167]. Simultaneously, a prevalent mutant large HBsAg protein with the deletion of amino acids 2 to 55 of the pre-S2 region, enhances the expression of pro-survival BCL-2 proteins; and BCL-2 contributes to 5-fluorouracil resistance in Huh-7 cells [168]. In addition, the HBV core protein inhibits apoptosis in HepG2 cells via the downregulation of Fas, p53 and FasL [169]. Another group shows that the core protein impairs the phosphorylation of mitogen-activated protein kinase kinase 7 (MKK7) by binding to its scaffold protein, RACK1, which consequently down-regulates JNK pathway and sensitizes HepG2 cells to TNF-α-induced apoptosis (Figure 1) [170].

## 4. Conclusions and Perspective

As an HCC-associated virus, HBV has drawn a lot of attention. To investigate the virus–cell interaction, a series of cell models are used in the experiments [171]. Primary human hepatocytes are the most physiologically relevant in vitro model for HBV infection with the natural viral receptor. However, the constraints of this cell model cannot be ignored, such as the limited span of life, limited sources and disparity from different donors. Huh-7 and HepG2 cell lines are commonly used in the studies, though tumor cells can only partially represent the physiological hepatic functions. To overcome the in vitro virus culture problem, hepatocyte cell lines stably transduced with HBV (HepAD38 and HepG2.2.15) are established as a source of HBV infectious particles [3,172,173,174]. However, the stable cell lines are not susceptible to HBV infection due to the lack of efficient receptors [175,176]. To obtain an efficient HBV replication, HepG2 cells stably expressing the HBV receptor (NTCP) have been established and might be useful for the basic research of HBV biology [3,174].

Although the HCC development is usually featured by the inhibition of apoptosis, the outcome of cell apoptosis is a result of a complex biological process, as there are different regulations of multiple cell signaling pathways by the various HBV proteins. Even the same apoptotic signaling pathway can be affected towards opposite consequences in the cells, such as the mitochondrial apoptotic pathway. This pathway can be activated by the BAX insertion into the mitochondrial membrane, which is driven by HBV (Figure 2). In contrast, this pathway can be inhibited by the recruitment of Parkin and Drp-1 during HBV infection (Figure 1). Activation of NF-κB signaling is a double-edged sword: promoting the expression of pro-survival genes to facilitate the cell proliferation and upregulating the expression of the death-associated receptor to sensitize cells to apoptotic stimuli. In the cell models with HBX overexpression, one of the potential considerations is that the protein overexpression level should mimic the “physiological level” in the HBV-infected patients, which can be achieved by optimization of transfection or a vector with a mild promoter [177]. In HepG2 cells, the HBX-deficient HBV replication can be rescued even when HBX is expressed at a very low level (beyond the detection limit of Western Blotting) [178]. The HBX functions identified in transiently transfected cells can be further assessed in HBV cell culture models. Therefore, the HBV effect on cell apoptosis varies depending on cellular context, different signaling pathways, HBV genotypes, protein mutations and possibly different clinical stages.

Technically, the cell culture models exhibit certain extent of defect due to its failure to mimic the host microenvironment. The mechanism of interaction between the virus and cell apoptosis should be further investigated under a more comprehensive situation. It is worthy to note that the frequently used cell line in the HBV biology study, Chang cells, are reported to have HeLa cell contamination at least in several clones [179]. Additional caution is needed when using this cell model in further studies.

Although apoptosis induction is widely considered as a positive strategy against cancer, the apoptotic cells are able to promote proliferation of the surrounding tumor cells by affecting the microenvironment [180,181,182]. In addition, there is no exact study of HBV effect on cell apoptosis in different clinical stages. Since HBV is frequently detected in clinical HCC cancers, it is more relevant that HBV/HBX accelerates cell transformation and facilitates apoptosis inhibition as a long-term effect. In most studies, HBV/HBX is shown to promote the anti-apoptotic proteins or inhibit the function of pro-apoptotic proteins. However, in some experiments, the higher level of ROS or the upregulated death associated receptors in the hepatocytes might be factors for apoptosis induction. In addition, co-effect of HBV/HBX levels in infected patients and the stimulation from the surrounding environment needs to be considered. We postulate there might be certain signaling-competing mechanism during the disease progression under the comprehensive effect of these myriad factors. The HBV levels in patients with acute or chronic HBV infection may be monitored and analyzed for correlation with outcomes in different clinical stages, if possible. Due to the limited host range of the virus, the establishment of an efficient animal model is experimentally important. According to the sequence analysis, duck hepatitis B virus and woodchuck hepatitis virus may be surrogate viruses in the HBV biological study. In addition, transgenic mice are frequently used in the pathogenesis and immune response studies of HBV infection. A more advanced experimental model is much needed to better elucidate the interaction mechanism between HBV and apoptosis.

## Abbreviations

TNF-α	Tumor necrosis factor alpha
TRAIL	TNF-related apoptosis-inducing ligand
APO2L	APO2 ligand
APO3L	APO3 ligand
BCL-2	B-cell lymphoma 2
BAK	BCL-2 homologous antagonist/killer
FADD	Fas-associated protein with death domain
DISC	death-inducing signaling complex
RIP	receptor-interacting protein
X-IAP	X-linked inhibitor of apoptosis protein
TRADD	TNFR type 1-associated DEATH domain protein
RIPK1	receptor-interacting serine/threonine-protein kinase 1
cIAP	cellular inhibitors of apoptosis
TRAF2/3/5/6	TNF receptor associated factors 2/3/5/6
cFLIP	Cellular FLICE-inhibitory protein
TCR	T cell receptor
BCR	B cell receptor
TAK1	Transforming growth factor beta-activated kinase 1
BAX	BCL-2-associated X protein
BAK	BCL-2 homologous antagonist/killer
APAF-1	apoptotic protease activating factor 1
ROS	reactive oxygen species
BAD	BCL-2-associated death promoter
BID	BH3 interacting-domain death agonist
SMAC	a second mitochondrial activator of caspases
AIF	apoptosis inducing factor
MOMP	mitochondrial outer membrane permeabilization
ncRNA	non-coding RNA
miR	microRNA
lncRNA	long coding RNA
HURP	Hepatoma upregulated protein
MPTP	mitochondrial permeability transition pore
PI3K	Phosphatidylinositol-4,5-bisphosphate 3-kinase
Akt	Protein kinase B
TGF-β	Transforming growth factor beta
SAPK	stress-activated protein kinase
BAFFR	TNF family receptor
LTβR	lymphotoxin β-receptor
RANK	receptor activator for nuclear factor κB
NIK	NF-kappa-B-inducing kinase
IKK	IκB kinase
PTEN	Phosphatase and tensin homolog
gp96	Heat shock protein 90kDa beta member 1
Mcl-1	Induced myeloid leukemia cell differentiation protein
p21	s cyclin-dependent kinase inhibitor 1
p27	Cyclin-dependent kinase inhibitor 1B
DR5	Death receptor 5/TRAIL receptor 2
IL-1/-6/-8	Interleukin-1/-6/-8
ER	endoplasmic reticulum
Mdm2	Mouse double minute 2 homolog
LC3 II	Microtubule-associated proteins 1A/1B light chain 3B
STAT3	Signal transducer and activator of transcription 3
CDC42	Cell division control protein 42 homolog
EGFP	Enhanced green fluorescent protein
CAT-1	Cationic amino acid transporter 1
Smad7	Mothers against decapentaplegic homolog 7
MLK3	Mitogen-activated protein kinase kinase kinase 3
MKK7	Dual specificity mitogen-activated protein kinase kinase 7
ERK	extracellular signal-regulated kinases
JNK	c-Jun N-terminal kinases
MEKK	MAP kinase kinase kinase
RACK	Receptor for activated C-kinase
Drp-1	Dynamin-1-like protein
Elk	ETS domain-containing protein
MAPKKs	MAP kinase cascades
NTCP	sodium taurocholate cotransporting polypeptide
